# Open-Set Radio Frequency Fingerprint Identification Method Based on Multi-Task Prototype Learning

**DOI:** 10.3390/s25175415

**Published:** 2025-09-02

**Authors:** Zhao Ma, Shengliang Fang, Youchen Fan

**Affiliations:** School of Aerospace Information, Space Engineering University, Beijing 101400, China; nuaamazhao@163.com (Z.M.); love1937@hgd.edu.cn (Y.F.)

**Keywords:** RF fingerprint identification (RFFI), open-set, prototype learning, extreme value theory (EVT)

## Abstract

Radio frequency (RF) fingerprinting, as an emerging physical layer security technology, demonstrates significant potential in the field of Internet of Things (IoT) security. However, most existing methods operate under a ‘closed-set’ assumption, failing to effectively address the continuous emergence of unknown devices in real-world scenarios. To tackle this challenge, this paper proposes an open-set radio frequency fingerprint identification (RFFI) method based on Multi-Task Prototype Learning (MTPL). The core of this method is a multi-task learning framework that simultaneously performs discriminative classification, generative reconstruction, and prototype clustering tasks through a deep network that integrates an encoder, a decoder, and a classifier. Specifically, the classification task aims to learn discriminative features with class separability, the generative reconstruction task aims to preserve intrinsic signal characteristics and enhance detection capability for out-of-distribution samples, and the prototype clustering task aims to promote compact intra-class distributions for known classes by minimizing the distance between samples and their class prototypes. This synergistic multi-task optimization mechanism effectively shapes a feature space highly conducive to open-set recognition. After training, instead of relying on direct classifier outputs, we propose to adopt extreme value theory (EVT) to statistically model the tail distribution of the minimum distances between known class samples and their prototypes, thereby adaptively determining a robust open-set discrimination threshold. Comprehensive experiments on a real-world dataset with 16 Wi-Fi devices show that the proposed method outperforms five mainstream open-set recognition methods, including SoftMax thresholding, OpenMax, and MLOSR, achieving a mean AUROC of 0.9918. This result is approximately 1.7 percentage points higher than the second-best method, demonstrating the effectiveness and superiority of the proposed approach for building secure and robust wireless authentication systems. This validates the effectiveness and superiority of our approach in building secure and robust wireless authentication systems.

## 1. Introduction

With the rapid advancement of the Internet of Things (IoT) and 5G technologies, the number of wireless devices is experiencing an explosive growth, presenting significant security challenges. Traditional cryptography-based security mechanisms are costly to deploy and complex to manage for resource-constrained IoT devices. Physical layer security (PLS) technology offers a novel approach to authentication by leveraging the physical characteristics of wireless channels and their device hardware [1,2]. Among these, radio frequency fingerprint identification (RFFI) technology—by analyzing the unique signal imprints generated by the inherent hardware imperfections of devices—enables precise device recognition and effectively mitigates attacks such as identity spoofing [3,4].

In recent years, deep learning (DL), with its powerful capabilities in automatic feature extraction and nonlinear modeling, has become the dominant technological paradigm in the RFFI domain [5]. Methods based on deep neural networks (DNNs) can directly learn high-dimensional, abstract fingerprint features from raw I/Q signals or their transformed domain representations, achieving near-perfect recognition accuracy in closed-set scenarios [6]. However, these successes often rely on a critical assumption: all devices encountered during the testing phase are known devices from the training phase. In real, dynamic wireless network environments, this assumption is clearly difficult to uphold. Unauthorized illegal devices or newly joined legitimate devices (collectively referred to as unknown devices) can appear at any time. When a closed-set model encounters unknown devices, its inherent SoftMax-based decision mechanism will forcibly map them to the most similar known class. This not only leads to classification errors but also creates a serious security vulnerability, potentially enabling attacks such as unauthorized access and identity masquerading [7,8].

Therefore, to advance RFFI technology toward practical applications, a paradigm shift from closed-set recognition to open-set recognition (OSR) is urgently needed. Open-set recognition requires models not only to accurately classify known device classes present in the training set but also to detect and reject unknown devices. Effectively implementing open-set RFFI faces two core challenges. The primary challenge lies in constructing a feature space that is amenable to open sets. This space must not only satisfy the “inter-class separability” required for traditional classification tasks but also exhibit high “intra-class compactness.” This means ensuring that samples belonging to the same known class have tightly clustered features, forming dense and clearly bounded clusters. This, in turn, helps define a maximally broad and distinct “open space” within the feature space, laying the foundation for reliably distinguishing known class samples from potential unknown class samples. Second, due to the complete absence of information about unknown class samples during the training phase, models must learn a decision boundary with strong generalization capabilities solely from known class data, enabling them to effectively handle an infinite array of potential unknown classes. Constructing such a boundary without prior knowledge of unknown classes represents the key challenge in open-set recognition [9].

To address these challenges, this paper proposes a novel open-set RFFI method based on Multi-Task Prototypical Learning (MTPL). We contend that a single cross-entropy classification optimization objective is insufficient to simultaneously meet the complex demands of open-set recognition concerning feature discriminability. Consequently, we designed a multi-task framework integrating classification, reconstruction, and prototypical learning. Specifically, the standard classification loss is responsible for separating feature clusters of different classes, ensuring their separability in the feature space. The reconstruction loss, implemented via an autoencoder structure, compels the model to learn the intrinsic manifold of the signals, thereby enhancing its ability to perceive unknown samples deviating from the normal distribution. Crucially, the prototypical loss directly minimizes the distance between samples and their respective class prototypes (feature centroids), achieving extreme intra-class compactness in the most straightforward manner. These three tasks are jointly optimized end-to-end via a hybrid loss function, systematically shaping a feature space characterized by a clear geometric structure and well-defined discriminative boundaries. For discrimination, we no longer rely on SoftMax probabilities. Instead, we compute the minimum distance between a sample’s features and all known class prototypes. We further incorporate extreme value theory (EVT) [10] to statistically model the distribution of these distances. This approach enables the scientific and adaptive determination of a decision threshold for distinguishing between known and unknown classes, avoiding the unreliability inherent in manual parameter tuning.

The main contributions of this paper are as follows:

(1) Design of the MTPL framework: We design the MTPL framework to jointly optimize classification loss, reconstruction loss, and prototypical loss while introducing learnable dynamic class prototypes. This framework not only effectively expands the inter-class distances between features of different classes but also forces features of the same class to aggregate tightly towards their class centers. Consequently, it constructs a feature space geometry that is both highly compact and discriminative, laying an ideal representational foundation for open-set recognition.

(2) Innovative application of EVT for open-set discriminative boundary construction: Distinct from traditional approaches reliant on empirical thresholds, we innovatively apply EVT to construct a statistically probabilistic open-set discriminative boundary. This is achieved by modeling the global tail of the distance distribution between all known class samples and their prototypes. This method adaptively calculates the probability of a sample belonging to a known class, enabling robust rejection of unknown devices.

(3) Comprehensive comparative experiments: We conducted comprehensive comparative experiments on public datasets. These include performance comparisons against six mainstream baseline methods and detailed ablation studies. The experimental results fully validate the superiority of the proposed method, particularly demonstrating significant improvements in AUROC—a key metric for open-set recognition. This work provides a new technical solution for the field of RF fingerprint identification.

The rest of this paper is organized as follows: Section 2 reviews related work. Section 3 introduces the problem formulation for open-set radio frequency fingerprint identification. Section 4 provides a detailed exposition of the proposed MTPL method. Section 5 presents and analyzes experimental results. Finally, Section 6 concludes this paper.

## 2. Related Works

This section aims to review the relevant research in the fields of RFFI and OSR. On this basis, it elaborates on the limitations of existing methods, thereby laying the foundation for the necessity and innovativeness of the new method proposed in this paper.

### 2.1. DL-Based RFFI Methods

RFFI technology provides a robust physical layer security mechanism for identity authentication of communication devices by analyzing the inherent and relatively stable radio frequency characteristics of device hardware over time. Early research mainly relied on manually designed features, such as constellations [11], higher-order moments [12], spectral characteristics [13], frequency offsets [14], etc., combined with traditional machine learning algorithms such as support vector machine (SVM) [15], K-nearest neighbors (KNN) [16], and other weak classifiers for classification. However, these methods often require strong domain knowledge to extract effective features, and their robustness and generalization ability are often limited by signal distortion [17] (such as multipath effects, frequency offsets, noise, etc.) and the growth in the number of devices [18].

In contrast, methods based on DNNs are capable of directly learning high-dimensional, abstract fingerprint features from raw I/Q signals or their transformed domain representations. In the closed-set scenario, such methods have demonstrated significant advantages, even achieving near-perfect recognition accuracy [8]. For instance, Riyaz et al. [19] pioneered the RFF-CNN1 model, composed of multiple layers of CNNs, which effectively identified Wi-Fi signals. Peng et al. [20] proposed converting I/Q data into Differential Constellation Trace Figures (DCTFs) before using CNNs to achieve accurate classification of five devices. Al-Shawabka et al. [21] explored the application of LSTM in RFFI and conducted experimental verification on a dataset of 100 LoRa devices. Liu et al. [22] further integrated the advantages of CNN and LSTM, designing a hybrid network model that significantly improved recognition accuracy.

### 2.2. Open-Set RFFI Method

As previously discussed, a robust RFFI system must not only correctly identify known devices but also effectively detect and reject unknown devices, achieving OSR. To address the OSR challenge, researchers have proposed various strategies, which can be broadly categorized as follows:

**Confidence-based methods** analyze the statistical distribution of model output probabilities. Leveraging the difference in prediction probabilities between known and unknown classes, they perform open-set recognition by setting a discrimination threshold. Representative approaches include comparing the sample’s maximum SoftMax probability (MSP) against a preset threshold [23] or calibrating the activation vector to improve unknown class detection, such as OpenMax [24]. Within the RFFI domain, related studies have explored detection mechanisms based on MSP or OpenMax [25] or combined fixed-threshold classifiers with calibration techniques to enhance open-set radio frequency fingerprint identification (OS-RFFI) performance [26,27]. However, these methods heavily depend on manually set thresholds and are often affected by the inherent overconfidence of deep models, making it difficult to establish a truly reliable decision boundary for unknown devices.

**Reconstruction-based methods** employ models like autoencoders to learn low-dimensional representations of input data and reconstruct it. Trained on known class data, the model learns their distribution characteristics. When presented with unknown class data, it typically generates higher reconstruction errors [28]. Building on this, references [29] proposed modeling a discrimination threshold based on reconstruction error; reference [30] designed a deep category reconstruction network and improved the activation vector calibration process of OpenMax; and reference [31] introduced diffusion models into OSR-RFFI, effectively distinguishing unknown classes by combining reconstruction error. Although reconstruction error can serve as a reference for identifying unknown devices, its practical effectiveness is often limited. The core reason lies in the frequent significant overlap between the reconstruction error distributions of known and unknown devices: On the one hand, unknown devices structurally similar to known devices may yield low reconstruction errors; on the other hand, noisy data or abnormal samples from known devices may instead exhibit high errors. This phenomenon of error confusion makes it difficult to set reliable and universal thresholds. Moreover, it poses severe challenges to practical applications in effectively distinguishing between known and unknown devices while avoiding misjudgment of known samples.

**Distance/metric learning-based methods** focus on optimizing the structure of the feature space. Their core hypothesis is that samples from known classes form compact clusters within this space, while samples from unknown classes lie far away from these clusters. Discrimination is performed by calculating the distance between a sample’s features and the centroids or prototypes of known classes. For instance, reference [32] proposes a class anchor clustering loss to enhance intra-class compactness, reference [33] combines center loss with classification loss to train the model and employs a fixed threshold to distinguish unknown classes, reference [34] integrates denoising autoencoders with deep metric learning for unknown class detection, reference [35] utilizes triplet loss to train the model and combines it with KNN for open-set discrimination and classification, and reference [36] introduces EVT on top of Feature Distance Measurement (FDM) to improve anomaly detection performance. A core challenge for such methods lies in constructing theoretically grounded open-set decision boundaries. While metric learning can effectively establish discriminative margins between known classes, it does not inherently model the overall boundary of the known feature space. Consequently, in practice, unknown samples are often rejected by setting distance-based thresholds, which are mostly determined through empirical methods rather than derived from the statistical properties of the data. This approach directly results in poor reliability of the final decision boundaries and weak generalization ability.

In summary, existing OSR methods face significant challenges when applied to RFFI: signal noise, distortion, and large intra-class variance lead to severe class confusion. This not only weakens the separability of known classes but also makes the effective rejection of unknown classes difficult. Consequently, learning a feature space characterized by “inter-class separability and intra-class compactness” and setting appropriate decision boundaries are crucial for the performance of open-set radio frequency fingerprint identification models.

## 3. Problem Definition

This paper primarily focuses on the problem of open-set radio frequency fingerprint recognition. Specifically, the task involves the following key elements: the training dataset $D_{\mathrm{train}\;}={\left\{\left(x_{i},y_{i}\right)\right\}}_{i=1}^{N}$ consists solely of samples from the set of known classes $C_{\mathrm{known}\;}=\{1,2,\ldots ,C\}$. Here, $x_{i}$ represents a preprocessed RF signal sample, $y_{i}\in C_{\mathrm{known}\;}$ is the corresponding device class label, and C denotes the total number of known classes.

The test dataset $D_{\mathrm{test}\;}$, however, comprises a more complex sample composition: it contains samples originating from both $C_{\mathrm{known}\;}$ and the set of unknown classes $C_{\mathrm{unknown}\;}$. Crucially, $C_{\mathrm{known}\;}$ and $C_{\mathrm{unknown}\;}$ are disjointed (i.e., $C_{\mathrm{known}}\cap C_{\mathrm{unknown}}=\emptyset$). Unknown classes refer to device types completely absent from the training data, with no prior information available to the model. The objective of this task is to train a classification function *f*. For any test sample $x_{\mathrm{test}\;}\in D_{\mathrm{test}\;}$, this function must achieve a dual functionality:

**Accurate identification of known classes:** If the sample’s true label $y_{\mathrm{test}\;}$ belongs to the known set $C_{\mathrm{known}\;}$ (i.e., $y_{\mathrm{test}\;}\in C_{\mathrm{known}}$), the model should output its correct class label: $f\left(x_{\mathrm{test}}\right)=y_{\mathrm{test}}$.

**Effective rejection of unknown classes:** If the sample’s true label $y_{\mathrm{test}\;}$ belongs to the unknown set $C_{\mathrm{unknown}\;}$ (i.e., $y_{\mathrm{test}\;}\in C_{\mathrm{unknown}}$), the model should classify it as ‘unknown’, rejecting its assignment to any known class: $f\left(x_{\mathrm{test}}\right)=\text{‘}\mathrm{unknown}\text{’}$.

The above objective can be formally expressed as follows:

(1) $$f\left(x_{\mathrm{test}}\right)=\left\{\begin{array}{cc} y_{\mathrm{test}\;} & \;\mathrm{if}\;y_{\mathrm{test}\;}\in C_{\mathrm{known}\;}\\ \;\mathrm{unknown}\; & \;\mathrm{if}\;y_{\mathrm{test}\;}\in C_{\mathrm{unknown}\;} \end{array}\right.$$

The core challenge lies in the requirement that the model must learn an effective decision boundary using only known class data ($D_{\mathrm{train}}$) for training. This boundary must not only ensure high discriminative power within the known classes for accurate classification but also reliably identify and reject unknown class samples that deviate from the distribution of known data. Essentially, this demands that the model learns a robust representation for “unknownness” without supervision from unknown class samples, thereby avoiding misclassifying unknown signals as the most similar or closest known class (i.e., generating false positives). Consequently, evaluating the performance of an open-set RF fingerprint recognition model requires a comprehensive consideration of its classification accuracy on known classes (closed-set accuracy) and its detection capability for unknown classes (typically measured by metrics such as the True Positive Rate for unknowns or detection rate and the False Positive Rate), seeking the optimal balance between these two aspects.

## 4. Proposed Method

To address the dual challenges of unknown device detection and accurate classification of known devices in open-set radio frequency fingerprint recognition, this section proposes an open-set recognition method based on MTPL. The core concept of this approach lies in the construction of a deep feature space that simultaneously exhibits intra-class compactness and inter-class separability through a well-designed multi-task learning framework. Furthermore, by integrating minimum prototype distance with EVT, the method establishes a statistical decision framework grounded in solid theoretical principles for open-set discrimination. This comprehensive solution effectively bridges the performance gap between closed-set classification and open-set recognition while also maintaining rigorous mathematical interpretability.

### 4.1. Method Overview

**Figure 1:**
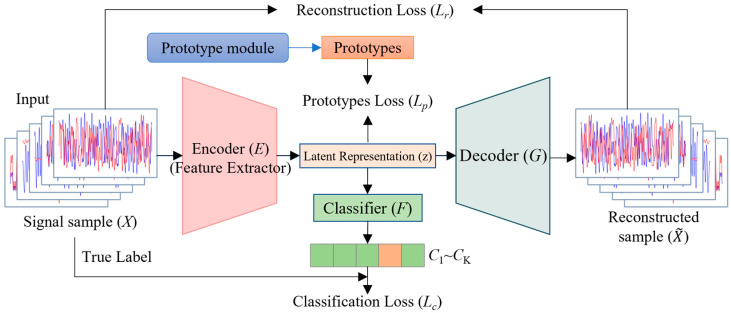
Framework structure of the proposed MTPL model.

As shown in the Figure 1, the overall framework of our method comprises an encoder *E_ϕ_*, a decoder *D_θ_*, and an auxiliary classifier *F_ψ_*. Its core operational mechanism is divided into two stages: feature space construction and open-set discrimination.

In the feature space construction stage, we adopt a multi-task joint optimization strategy. Firstly, by combining standard classification loss with a learnable prototype loss, we drive the encoder to learn highly discriminative features. The classification loss ensures the separability of features across classes, while the prototype loss explicitly pulls features of intra-class samples toward their class-specific prototypes in the embedding space, thus forming compact intra-class clusters. This compactness provides a crucial prior structure for distinguishing known from unknown signals. Secondly, we introduce a reconstruction loss, requiring that the features extracted by the encoder be reconstructed to the original signal as losslessly as possible by the decoder. This task serves as a regularization technique, ensuring that the features are not only discriminative but also information-complete, effectively preventing the model from discarding the intrinsic physical properties of the signal in pursuit of classification accuracy, thereby enhancing sensitivity to out-of-distribution (unknown) signals.

In the open-set discrimination stage, we move away from methods that rely on empirically set decision thresholds. Instead, we leverage extreme value theory (EVT) to statistically model the tails of the prototype distance distributions for all known classes. Specifically, we calculate the distance between each training sample’s feature and its corresponding class prototype. We then fit the extreme portions of these distances using the Generalized Pareto Distribution (GPD). This provides us with a probabilistic model describing how far a sample from a known class should be from its class center. When a test sample arrives, we compute its minimum distance to all class prototypes. Utilizing the fitted GPD model, we perform a statistical hypothesis test at a pre-defined significance level to determine whether to assign the sample to a known class or reject it as an unknown device.

Through this two-stage design, we ensure that the feature space is first optimized to an ideal state, and then discrimination is performed using a statistical decision framework specifically designed for open-set scenarios.

### 4.2. Model Optimization Objectives

To achieve the aforementioned feature space, we design a total optimization objective comprising three loss functions. Through an end-to-end training approach, we simultaneously optimize the encoder, decoder, classifier, and the set of prototypes. The total loss function is defined as

(2) $$L_{\mathrm{total}\;}=L_{c}+\lambda_{r}L_{r}+\lambda_{p}L_{p}$$

wherein $L_{c}$, $L_{r}$ and $L_{p}$ represent classification loss, prototype loss, and reconstruction loss, respectively, while $\lambda_{r}$ and $\lambda_{p}$ are super parameters used to balance various losses.

#### 4.2.1. Classified Loss

For a given input signal $x_{i}$, the encoder $E_{\phi }$ extracts a feature vector $z_{i}=E_{\phi }\left(x_{i}\right)$ of dimension $D_{\mathrm{feat}\;}$. The classifier $F_{\psi }$ receives this feature and outputs logits corresponding to the *K* known classes. After activation by the SoftMax function, we obtain the probability distribution $p_{i}$. We employ the standard cross-entropy loss to optimize the classification performance:

(3) $$L_{C}=-\frac{1}{B}\sum_{i=1}^{B}\sum_{k=1}^{K}y_{i,k}\log\left(p_{i,k}\right)$$

Here, *B* is the batch size, and $y_{i,k}$ is the one-hot encoding indicating whether sample *i* belongs to class *k*. This loss directly drives the model to learn features that can clearly distinguish between different known classes.

#### 4.2.2. Prototype Loss

To enhance the intra-class compactness of features, we introduce a learnable prototype vector $c_{k}$ of dimension $D_{\mathrm{feat}\;}$ for each known class *k*. All *K* prototypes form the prototype matrix $C\in {\mathbb{R}}^{K\times D_{\mathrm{feat}}}$. The prototype loss is defined as the mean squared Euclidean distance between each sample’s feature in the batch and its corresponding class prototype:

(4) $$L_{p}=\frac{1}{B}\sum_{i=1}^{B}{\left\|\begin{array}{c} E_{\phi }\left(x_{i}\right)-c_{y_{i}} \end{array}\right\|}_{2}^{2}=\frac{1}{B}\sum_{i=1}^{N}{\left\|\begin{array}{c} z_{i}-c_{y_{i}} \end{array}\right\|}_{2}^{2}$$

Here, $z_{i}$ is the feature vector of sample $x_{i}$, and $c_{y_{i}}$ is the learnable prototype corresponding to its true class $y_{i}$. During the backpropagation process, this loss not only updates the parameters *ϕ* of the encoder to pull $z_{i}$ towards $c_{y_{i}}$ but also updates the prototype $c_{y_{i}}$ itself, moving it towards the “centroid” of all sample features for that class.

#### 4.2.3. Reconstruction Loss

The task of the decoder $D_{\theta }$ is to reconstruct the feature vector $z_{i}$ back to the original signal ${\hat{x}}_{i}=D_{\theta }\left(z_{i}\right)$. We use the mean squared error (MSE) to measure the difference between the original signal $x_{i}$ and the reconstructed signal ${\hat{x}}_{i}$:

(5) $$L_{r}=\frac{1}{B}\sum_{i=1}^{B}{\left\|\begin{array}{c} x_{i}-{\hat{x}}_{i} \end{array}\right\|}_{2}^{2}$$

By minimizing this loss, the encoder is encouraged to capture the primary variation modes essential for reconstruction, effectively learning the low-dimensional manifold of known class signals. When an unknown device signal is input, its pattern typically deviates from all known classes, making it difficult for the model to reconstruct effectively, thus resulting in a larger reconstruction error. This provides auxiliary information for open-set recognition.

We integrate the aforementioned three loss functions into a unified optimization framework, enabling end-to-end optimization of model parameters and prototypes through the backpropagation algorithm. The complete training procedure is formalized in Algorithm 1.
**Algorithm 1:** MTPL Model Training and Prototype Learning
**Require:** Training dataset $D_{\mathrm{train}\;}={\left\{\left(x_{i},y_{i}\right)\right\}}_{i=1}^{N}$;
Number of known classes K;Loss weights *λ*_p_, *λ*_r_;Batch size B;Number of training epochs, *E*_p_.**Ensure:** Optimized model parameters (ϕ,θ,ψ);Prototype set $C={\left\{c_{k}\right\}}_{k=1}^{K}$.1Initialize parameters for the encoder $E_{\phi }$$,\;\mathrm{decoder}\;D_{\theta }$, and classifier $F_{\psi }$.2Initialize the learnable prototype matrix $C\in {\mathbb{R}}^{K\times D_{\mathrm{feat}}}$ using Gaussian random initialization.3**For** epoch = 1 to *E_p_*
**do:**4 **for** mini-batch $\left\{\left(x_{b},y_{b}\right)\right\}$ from $D_{\mathrm{train}}$
**do**:5
 **//Forward propagation**
6  Latent features: $z_{b}\leftarrow E_{\phi }\left(x_{b}\right)$;7  Reconstructed signal: ${\hat{x}}_{b}\leftarrow D_{\theta }\left(z_{b}\right)$;8  Classification logits: ${\mathrm{logits}}_{b}\leftarrow F_{\psi }\left(z_{b}\right)$;9  Classification probabilities: $p_{b}\leftarrow \mathrm{Softmax}({\mathrm{logits}}_{b})$.10
 **//Multi-Task Loss Calculation**
11  Classification loss: $L_{c}\leftarrow CrossEntropyLoss\left(p_{b},y_{b}\right)$;12  Prototype loss: $L_{p}\leftarrow \frac{1}{B}\sum_{i=1}^{B}{\left\|\begin{array}{c} z_{b,i}-c_{y_{b,i}} \end{array}\right\|}_{2}^{2}$;13  Reconstruction loss: $L_{r}\leftarrow \frac{1}{B}\sum_{i=1}^{B}{\left\|\begin{array}{c} x_{b,i}-{\hat{x}}_{b,i} \end{array}\right\|}_{2}^{2}$;14  Total loss: $L_{total}\leftarrow L_{c}+\lambda_{p}L_{p}+\lambda_{r}L_{r}$.15
 **//Backward Propagation and Parameter Update**
16  Compute gradients of $L_{total}$ with respect to all learnable parameters $\left(\phi ,\theta ,\psi ,C\right)$;17  Update parameters $\left(\phi ,\theta ,\psi ,C\right)$ using an optimizer.18
 **end for**
19**End for**20**Return** Trained parameters (ϕ,θ,ψ) and prototype set C


It should be further noted that although the MTPL framework introduces a decoder for reconstruction and learnable class prototype parameters during the joint optimization of multiple tasks—resulting in increased overhead in gradient computation and backpropagation during the training phase—only the encoder and class prototypes need to be retained to achieve open-set discrimination of signals during the inference phase after training is completed. Therefore, in the subsequent deployment phase during inference, the model introduces almost no additional computational burden, and its inference efficiency is almost entirely determined by the network structure of the encoder.

### 4.3. Open-Set Discrimination with EVT

In the feature space constructed by our method, known class samples form compact clusters around their respective class prototypes. Based on this structure, a reliable mechanism is required to distinguish between known and unknown samples. A common approach is to set a global distance threshold. However, the performance of such methods is highly dependent on the threshold selection and lacks adaptability to data distribution shifts. An unknown device, being fundamentally different from any known class, should produce a feature vector distant from all class prototypes. This large distance can be modeled as an extreme value, making EVT a natural fit for this problem. Therefore, we introduce EVT to build a more principled discrimination model. EVT, a branch of applied mathematics, is used for analyzing and modeling the statistical behavior of extreme deviations in random variables.

One of its core theorems, the Pickands–Balkema–de Haan theorem, states that for a sufficiently high threshold *ω*, the distribution of excesses over this threshold (i.e., *d* − *ω*) can be approximated by the Generalized Pareto Distribution (GPD).

The cumulative distribution function (CDF) of the GPD is given by

(6) $$F\left(v;\xi ,\sigma \right)=\left\{\begin{array}{ll} 1-{\left(1+\frac{\xi v}{\sigma }\right)}^{-1/\xi } & \;\mathrm{if}\;\xi \neq 0\\ 1-e^{-v/\sigma } & \;\mathrm{if}\;\xi =0 \end{array}\right.$$

Here, *y* represents the exceedance over the threshold (i.e., *v* = *d* − *ω*, where *d* > *ω*), *σ* > 0 is the scale parameter, and *ξ* is the shape parameter.

**Figure 2:**
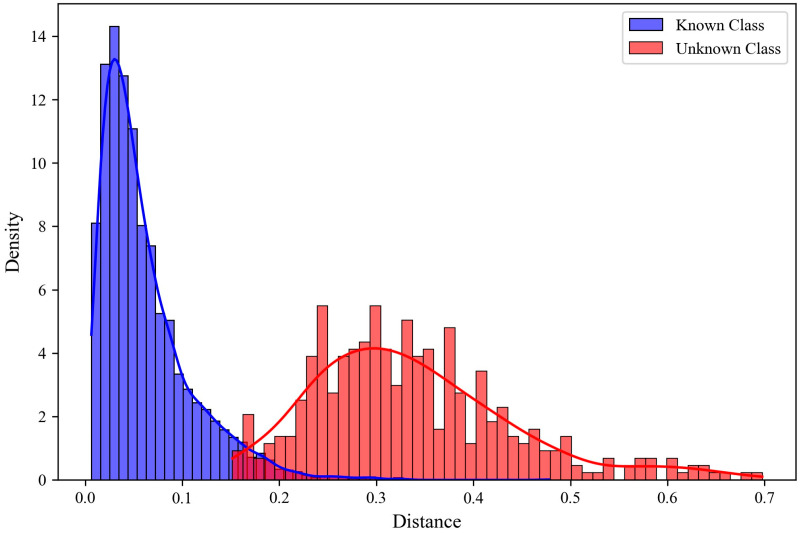
Distribution of prototype distances for known and unknown classes.

As shown in Figure 2, we present the distribution of minimum prototype distances for samples from known and unknown classes. Analysis of these distance distributions reveals a clear distinction between samples belonging to known classes and those belonging to unknown classes. We employ the GPD to model the tail of the prototype distance distribution for known class samples, thereby transforming the open-set discrimination problem into a statistical hypothesis testing problem. This process involves two stages: offline modeling and online decision-making.

#### 4.3.1. Tail Modeling Based on Prototype Distances

Following the completion of model training, the objective of this stage is to utilize the training data to fit a GPD model that describes the tail distribution of global intra-class distances. This process is essentially an estimation of the GPD parameters.

(1) Global Intra-Class Distance Aggregation

We first collect the fundamental data for modeling. By iterating through all samples $\left(x_{i},y_{i}\right)$ in the training set $D_{train}$, we compute their characteristic feature vectors $z_{i}$. The squared Euclidean distance $d_{i}={\left\|\begin{array}{c} z_{i}-c_{y_{i}} \end{array}\right\|}_{2}^{2}$ between $z_{i}$ and its corresponding class prototype $c_{y_{i}}$ is calculated. All these distances collectively form the global distance score set $D_{\mathrm{scores}}$.

(2) GPD Parameter Estimation

The establishment of a GPD model requires estimating three parameters from the data: the threshold *ω*, the scale parameter *σ*, and the shape parameter *ξ*.

**Threshold selection:** The choice of the threshold requires a balance between model bias and estimation variance. If the threshold is too low, it may violate the asymptotic assumptions of EVT. If the threshold is too high, the tail data used for fitting will be too sparse, leading to increased estimation variance. A common approach is to select a high quantile of $D_{\mathrm{scores}}$ as the threshold $\omega_{\mathrm{global}}$.

**Scale and shape estimation:** After determining the threshold $\omega_{\mathrm{global}}$, we select all distances exceeding this threshold and compute their excesses to form the tail dataset $T_{\mathrm{global}}=\left\{d_{i}-\omega_{\mathrm{global}}|d_{i}\in D_{\mathrm{scores}},\;d_{i}>\omega_{\mathrm{global}\;}\right\}$. Subsequently, we employ the Maximum Likelihood Estimation (MLE) method to estimate the parameters σ and ξ. This method aims to find a set of parameters $\left(\hat{\sigma },\hat{\xi }\right)$ that maximizes the probability of observing the current tail dataset $T_{\mathrm{global}}$. The log-likelihood function of the GPD is given by

(7) $$L\left(\sigma ,\xi ;T_{\mathrm{global}\;}\right)=-m\log\sigma -\left(1+\frac{1}{\xi }\right)\sum_{i=1}^{m}\log\left(1+\frac{\xi v_{i}}{\sigma }\right)$$

where *m* is the size of the tail dataset, and $v_{i}\in T_{global}$. This function can be solved via numerical optimization methods to obtain the estimated parameters $\hat{\sigma }$ and $\hat{\xi }$.

Through the steps above, we obtain a parameter-determined GPD model, $G_{\mathrm{global}\;}\left(\omega_{\mathrm{global}\;},\hat{\sigma },\hat{\xi }\right)$, which characterizes the tail statistical properties of the global intra-class distances. The process of offline fitting the global GPD model, encompassing distance aggregation and parameter estimation, involves two core steps. The detailed implementation of this process is elaborated in Algorithm 2.
**Algorithm 2:** Offline GPD Model Fitting.
**Require:** Trained encoder parameters ϕ and prototype set $C={\left\{c_{k}\right\}}_{k=1}^{K}$;Complete training dataset $D_{train}={\left\{\left(x_{i},y_{i}\right)\right\}}_{i=1}^{N}$;Quantile *q* for determining the tail threshold (in this paper, *q* = 0.9).
**Ensure:** Parameters for the global GPD model: threshold $\omega_{global}$, scale parameter σ, shape parameter ξ.1Initialize an empty list for distance scores: $D_{scores}\leftarrow []$.2**Global Intra-class Distance Aggregation**3**For** each sample $\left(x_{i},y_{i}\right)$ in $D_{train}$
**do**:4 Extract feature: $z_{i}\leftarrow E_{\phi }\left(x_{i}\right)$;5 Retrieve the corresponding class prototype: $c_{y_{i}}\leftarrow C\left[y_{i}\right]$;6 Compute the squared Euclidean distance: $d_{i}\leftarrow {\left\|\begin{array}{c} z_{i}-c_{y_{i}} \end{array}\right\|}_{2}^{2}$;7 Append distance to the list: $d_{i}\to D_{\mathrm{scores}}$.8**End for**9**GPD Parameter Estimation**10 (a) Determine the Tail Threshold11  $\omega_{\mathrm{global}}\leftarrow Quantile\left(D_{\mathrm{scores}},q\right)$;12 (b) Extract Tail Data (Exceedances)13  $T_{\mathrm{global}}\leftarrow \left\{d-\omega_{\mathrm{global}}|d\in D_{\mathrm{scores}}\right.,\;\left.d>\omega_{\mathrm{global}}\right\}$;14 (c) Fit GPD Model using Maximum Likelihood Estimation15  
$\left(\sigma ,\xi \right)\leftarrow FitGPD\_MLE\left(T_{\mathrm{global}\;}\right)$
**.**
16**Return** $\omega_{\mathrm{global}},\sigma ,\xi$

#### 4.3.2. Online Identification and Decision-Making

Upon receiving a test sample *x*_test_, we utilize the fitted GPD model to perform online decision-making.

(1) Computation of Test Statistic

First, we extract the feature vector $z_{test}$ of the test sample. Then, we compute the minimum squared Euclidean distance between $z_{test}$ and all known class prototypes. This minimum squared distance serves as the test statistic: $d_{\min}=\underset{k\in \{1,\ldots ,N\}}{\min}{\left\|\begin{array}{c} z_{test}-c_{k} \end{array}\right\|}_{2}^{2}$. Simultaneously, we record the index $\hat{k}$ of the prototype corresponding to this minimum distance.

(2) Hypothesis Testing and *p*-value Computation

We formalize the decision process as a statistical hypothesis test:

- Null hypothesis (*H*_0_): The test sample *x*_test_ belongs to one of the known classes.
- Alternative hypothesis (*H*_1_): The test sample *x*_test_ belongs to an unknown class.

The decision rule is based on the relationship between *d*_min_ and the global threshold $\omega_{\mathrm{global}}$:

If $d_{\min}\leq \omega_{\mathrm{global}}$, it indicates that this distance has not entered the tail distribution region, representing typical in-class behavior. Therefore, we do not reject the null hypothesis *H*_0_.

If $d_{\min}>\omega_{\mathrm{global}}$, it indicates that the observation falls into the tail distribution, and we need to evaluate its extremity. We compute the *p*-value (probability value). The *p*-value is the probability of observing a distance that is more extreme (larger) than the current *d*_min_, given that the null hypothesis *H*_0_ is true. This value can be calculated from the survival function (1-CDF) of the GPD model:

(8) $$p\mathrm{-value}=P\left(D>d_{\min}|D>\omega_{\mathrm{global}}\right)={\left(1+\frac{\hat{\xi }\left(d_{\min}-\omega_{\mathrm{global}}\right)}{\hat{\sigma }}\right)}^{-1/\hat{\xi }}$$

(3) Final Decision

We compare the computed *p*-value with a pre-defined significance level, *δ_w_* (This article is set to 0.01).

- If *p*-value < $\delta_{w}$, it suggests that, under the assumption that *H*_0_ is true, *d_min_* is a low-probability event. Therefore, we reject the null hypothesis *H*_0_ and classify the sample as “Unknown.”
- If *p*-value ≥ $\delta_{w}$, it indicates that there is insufficient statistical evidence to reject the null hypothesis. Thus, we accept *H*_0_ and classify the sample into the candidate class $\hat{k}$.

This procedure replaces empirical threshold selection with a standard statistical inference framework, thereby providing stronger theoretical backing and robustness for open-set decision-making. We summarize this complete online identification process—from computing the minimum prototype distance of the test sample to making the classification or rejection decision through hypothesis testing—in Algorithm 3.
**Algorithm 3:** Online Open-Set Recognition
**Require:**Test sample: $x_{\mathrm{test}}$;Trained encoder parameters ϕ and prototype set $C={\left\{c_{k}\right\}}_{k=1}^{K}$;Global GPD model parameters: threshold $\omega_{\mathrm{global}}$, scale σξ (obtained from Algorithm 2);Significance level $\;\delta_{w}$ for decision-making (e.g., 0.01).**Ensure:**Predicted label $\hat{y}\in \{1,\ldots ,K\}\cup \{‘\mathrm{Unknown}’\}$.1**//Compute Test Statistics:**2 Extract feature: $z_{test}\leftarrow E_{\phi }\left(x_{\mathrm{test}}\right)$;3 Compute distances to all prototypes: $d_{k}\leftarrow {\left\|\begin{array}{c} z_{test}-c_{k} \end{array}\right\|}_{2}^{2}\;for\;k=1,\ldots ,K$;4 Find the minimum distance and its corresponding candidate class:5$d_{min}\leftarrow \min\left({\left\{d_{k}\right\}}_{k=1}^{K}\right)$$,\;\hat{k}\leftarrow {\mathrm{argmin}}_{k}\left({\left\{d_{k}\right\}}_{k=1}^{K}\right)$.6**//Decision Based on Extreme Value Model**7 **If**
$d_{\min}\leq \omega_{\mathrm{global}}$
**then**:8  $d_{\min}$ outside tail distribution, classify as known class: $\hat{y}\leftarrow \hat{k}$.9 
**else**
10  $d_{\min}$ falls into the tail distribution, perform hypothesis testing:11  //(a) Compute *p*-value: 12$p-\mathrm{value}\leftarrow {\left(1+\frac{\xi (d_{min}-\omega_{global})}{\sigma }\right)}^{-1/\xi }$;13  //(b) Compare with Significance Level:14  **if**
$p-\mathrm{value}<\delta_{w}$
**then**:15   Low probability event occurs; reject null hypothesis:16classify as unknown: $\hat{y}\leftarrow \text{‘}\mathrm{Unknown}’.$
17  
**else**
18   Null hypothesis cannot be rejected:
         classify as known class: $\hat{y}\leftarrow \hat{k}$.19  
**end if**
20 
**End if**
21**Return** $\hat{y}$

## 5. Experimental Verification and Analysis

### 5.1. Dataset Introduction

This paper employs a publicly available Wi-Fi dataset [37] for experimental validation. The dataset was collected using a USRP B210 receiver capturing signals from 16 USRP X310 radio transmitters during wireless transmissions. All transmitters generated IEEE 802.11a standard-compliant frames using the MATLAB WLAN System Toolbox. The receiver sampled the incoming Wi-Fi signals at a center frequency of 2.45 GHz with a sampling rate of 5 MS/s. Over 20 million I/Q samples were collected indoors per transmitter. After normalizing the I/Q signals, we partitioned the dataset into samples. Each sample has a fixed length of 4800 I/Q points, resulting in 12,000 samples per device. For the experiments in this section, we designate 10 classes as known devices and the remaining 6 classes as unknown devices.

### 5.2. Network Architecture and Experimental Setup

In this paper, both the encoder and decoder employ a complex-valued neural network structure recognized for its excellent performance, while the classifier adopts a simple fully connected structure. The detailed network architectures are shown in Table 1.

All experiments in this study were conducted on a hardware platform equipped with an Intel^®^ Core™ i7-10700k CPU @ 3.80 GHz and an NVIDIA GeForce RTX 3090 GPU, using Python 3.8 and the PyTorch 1.13.0 deep learning framework. During model training, the Adam optimizer was uniformly adopted with an initial learning rate of 0.001. A ReduceLROnPlateau dynamic learning rate scheduling strategy was employed, where the learning rate was reduced by 50% if no improvement in validation loss was observed for 20 consecutive epochs. The total training epochs for all models were set to 100, with a fixed batch size of 128.

For the proposed method, the hyperparameters weighting the prototype loss and reconstruction loss, denoted as *λ_p_* and *λ_r_*, were set to 0.05 and 0.1, respectively. In the open-set discrimination stage, the tail quantile *q* used for fitting the GPD model was set to 0.1, and the significance level *δ_w_* for the final decision was set to 0.01. To ensure fairness in comparative experiments, the discrimination thresholds for all baseline methods relying on empirical thresholds were determined by searching for optimal values on the validation set.

### 5.3. Evaluation Metrics

To comprehensively assess the model’s performance, we simultaneously consider its classification capability on known classes and its discrimination ability on unknown classes. Let *TP*_s_, *TN*_s_, *FP*_s_, and *FN*_s_ denote the true positives, true negatives, false positives, and false negatives, respectively, for the *s*-th known device.

**Overall accuracy (*P_Acc_*):** This metric measures the model’s comprehensive ability to correctly classify all known devices and correctly reject unknown devices. It is defined as

(9) $$P_{Acc}=\frac{\sum_{s=0}^{K-1}\left(TP_{s}+TN_{s}\right)}{\sum_{s=0}^{K-1}\left(TP_{s}+TN_{s}+FP_{s}+FNs\right)}\times 100\%$$

**ROC curve and AUROC:** The Receiver Operating Characteristic (ROC) curve (also known as the subject characteristic curve) is an important tool for visualizing the classifier’s performance changes across different discrimination thresholds. It is plotted with the True Positive Rate (TPR) on the *y*-axis and the False Positive Rate (FPR) on the *x*-axis. In this task, TPR quantifies the model’s ability to correctly identify known class samples, while FPR measures the proportion of unknown class samples that are incorrectly identified as known classes. Their definitions are as follows:

(10) $$\mathrm{TPR}=\frac{\sum_{s=0}^{K-1}TP_{s}}{\sum_{s=0}^{K-1}\left(TP_{s}+FN_{s}\right)}$$

(11) $$\mathrm{FPR}=\frac{\sum_{s=0}^{K-1}FP_{s}}{\sum_{s=0}^{K-1}\left(FP_{s}+TN_{s}\right)}$$

Since the ROC curve itself is difficult to directly compare the advantages of different models, we adopt the Area Under the ROC Curve (AUROC) as the core evaluation metric for open-set performance. AUROC is a value between 0 and 1 that quantifies the model’s overall discrimination ability across all possible thresholds. A higher AUROC value, closer to 1, indicates better performance in distinguishing between known and unknown classes and less sensitivity to threshold selection.

### 5.4. Comparison Methods

To validate the effectiveness of our proposed method, we selected five representative open-set recognition baseline methods for comparative experiments. For fairness and a direct comparison of the core open-set discrimination mechanisms, the backbone feature extractor (encoder) for all compared methods was kept identical to our proposed approach. The core ideas/training objectives and open-set discrimination mechanisms of these baseline methods are summarized in Table 2.

### 5.5. Experimental Results and Analysis

#### 5.5.1. Unknown Class Detection Performance Evaluation

Table 3 presents the performance of various methods on the AUROC metric under different configurations of unknown device quantities (from *N* = 1 to *N* = 6). As can be seen, our method, MTPL, achieved the optimal AUROC value across all configurations, with an average AUROC reaching as high as 0.9918. This demonstrates MTPL’s strong generalization capability and robustness in distinguishing between known and unknown devices.

As the number of unknown devices increases (e.g., *N* = 4, 5, 6), the traditional SoftMax threshold method experiences a drastic performance decline, with AUROC values merely ranging between 0.6 and 0.7. The OpenMax method, although it calibrates activation scores using extreme value theory, also performs poorly in these scenarios. Its AUROC values fluctuate significantly and even drop below SoftMax when *N* is between 2 and 4.

The MLOSR method exhibits excellent performance when the number of unknown devices is small (*N* = 1, 2), with AUROC values close to 0.91. However, its performance degrades as the number of unknown devices increases, notably dropping to 0.8658 at *N* = 6. SR2CNN, as a multi-task learning approach, performs well when the number of unknown devices is small (AUROC > 0.93), but its AUROC also shows a certain degree of decline when facing a larger number of unknown devices. TripletNet demonstrates strong capability in terms of AUROC through metric learning, with AUROC values exceeding 0.97 for *N* = 1 through *N* = 5, only slightly decreasing to 0.9523 at *N* = 6.

In contrast, our MTPL method consistently demonstrates stable and superior performance across various numbers of unknown devices. Under all experimental settings, MTPL’s AUROC values remain above 0.96 and, in most cases, approach or exceed 0.99. This strongly validates that the feature space constructed through our multi-task collaborative learning effectively mitigates interference from unknown devices, thereby enabling precise detection of unknown entities. Its superior performance is attributed to the intra-class compactness enforced by the prototype loss and the signal’s intrinsic information preserved by the reconstruction loss.

#### 5.5.2. Classification Performance Evaluation

This section aims to evaluate the overall performance of different open-set radio frequency fingerprinting methods. As defined in Section 5.3, this performance comprehensively integrates the ability to correctly identify known classes (True Positive Rate) with the ability to correctly reject unknown classes (True Negative Rate), measured by average recognition accuracy. The experiments were conducted on the Wi-Fi dataset, varying the number of unknown device classes (*N*) to observe the change in average recognition accuracy for each method under different proportions of unknown samples introduced (see Table 4).

In the closed-set scenario (*N* = 0, meaning only known classes exist), all methods achieve an average recognition accuracy close to 100%. However, as the number of unknown classes increases, the ability of each method to maintain this overall recognition accuracy, as defined previously, begins to diverge.

SoftMax, serving as a baseline, shows a rapid decline in its average recognition accuracy as the number of unknown classes (*N*) increases, dropping from 99.85% at *N* = 0 to 66.23% at *N* = 6. This directly exposes its vulnerability in open-set scenarios, where it struggles to effectively distinguish unknown samples, leading to a large number of misclassifications and significantly reducing the overall recognition accuracy.

Methods like MLOSR and SR2CNN attempt to enhance model sensitivity to unknown samples by incorporating reconstruction error or combining it with center loss. MLOSR’s performance in average recognition accuracy degrades rapidly with the increase in *N*, particularly at *N* = 6, where it drops to 62.32%, indicating a poor balance between distinguishing unknown samples and maintaining discriminative power for known classes. SR2CNN’s average recognition accuracy exhibits significant fluctuations; although its AUROC value (average 0.9434) is relatively good, its discriminative mechanism shows deficiencies in accurately classifying known devices, affecting the stability of overall recognition accuracy.

The OpenMax method calibrates the SoftMax output using extreme value theory, aiming to improve unknown sample detection capabilities. Its average recognition accuracy shows some improvement compared to SoftMax, but its performance becomes less stable as *N* increases, dropping to 78.39% at *N* = 6.

TripletNet, through metric learning, demonstrates significant advantages in average recognition accuracy (averaging 96.00%) and maintains over 96% from *N* = 1 to *N* = 5, fully validating the effectiveness of metric learning in enhancing overall robustness in open-set scenarios. Its AUROC value (average 0.9746) is also outstanding. However, at *N* = 6, its average recognition accuracy drops to 91.48%, suggesting potential limitations of its KNN discriminative mechanism when dealing with complex distributions.

Our MTPL method consistently exhibits the most stable and highest overall recognition accuracy across all tested scenarios. As the number of unknown classes (*N*) increases, its average recognition accuracy slightly decreases from 98.53% at *N* = 1 to 92.41% at *N* = 6, but it consistently maintains a very high level, significantly outperforming all comparative methods. This superior performance is attributed to the synergistic effect of its multi-task learning framework. By integrating prototype loss, reconstruction loss, and the EVT-driven adaptive discriminative mechanism, MTPL effectively avoids interference from unknown samples, maintaining leading overall recognition accuracy across all unknown class configurations and achieving a perfect balance between accurate identification of known classes and effective detection of unknown classes.

#### 5.5.3. Feature Visualization Experiment

**Figure 3:**
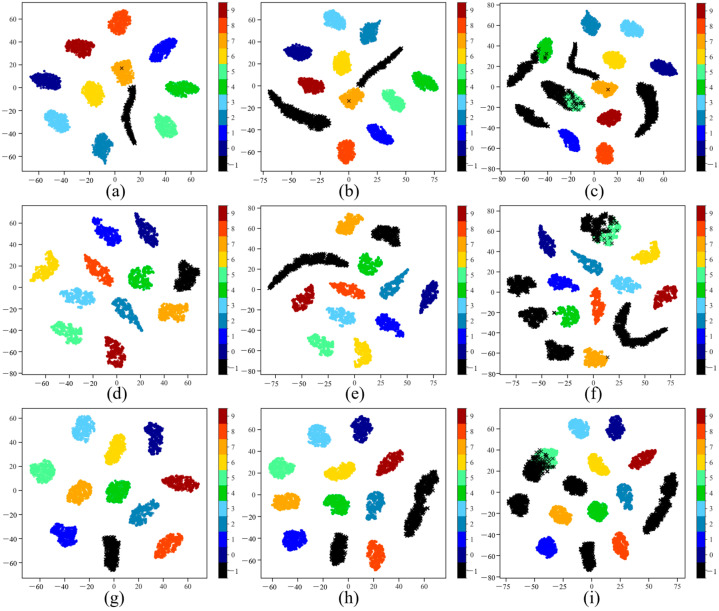
T-SNE visualization of feature distributions for different methods with *K* = 10 and *N* = 1, 3, 6. (**a**–**c**) SR2CNN; (**d**–**f**) TripletNet; (**g**–**i**) MTPL.

To investigate the distribution characteristics of different open-set recognition methods in the feature space, we utilized t-SNE to perform dimensionality reduction and visualize the extracted high-dimensional features. The experimental setup included 10 known classes (*K* = 10), and scenarios with three different quantities of unknown classes (*N* = 1, *N* = 3, *N* = 6). For comparison, we selected SR2CNN and TripletNet, which demonstrated the best performance, alongside our proposed MTPL method. Figure 3 presents the feature distributions of each method under these scenarios.

The visualization results reveal distinct performance differences among the methods in the feature space. SR2CNN forms some clusters between known classes, but the compactness of these clusters and the separation between classes are relatively low. This is particularly evident when the number of unknown classes increases, leading to blurred boundaries between classes, and with some unknown samples appearing mixed within the known class clusters. These feature distribution characteristics indicate that although SR2CNN possesses a certain capability for unknown sample detection (as suggested by its high AUROC values), its ability to finely distinguish known classes is significantly affected by interference from unknown samples.

TripletNet, through metric learning, forms more compact known class clusters in the feature space, with relatively clear boundaries between classes. When the number of unknown samples is small, these unknown samples are primarily distributed in the regions outside the known class clusters, demonstrating good separation of known/unknown classes. This aligns with TripletNet’s excellent performance in known class accuracy and AUROC values. However, when the number of unknown classes increases to *N* = 6, some unknown samples begin to appear at the edges of the known class clusters, indicating potential robustness limitations of its discriminative mechanism when facing more complex distributions.

In contrast, our MTPL method exhibits the most optimal structural characteristics in feature visualization. Across all scenarios with varying numbers of unknown classes, the known class clusters learned by MTPL are highly compact, with clear boundaries between classes. More importantly, unknown samples are effectively isolated outside the known class clusters, showing minimal internal intrusion. These feature distributions suggest that MTPL, through its multi-task learning framework, can learn feature representations that are highly sensitive to unknown samples while maintaining strong discriminative power for known classes. The prototype loss promotes intra-class compactness and clear inter-class separation, while the reconstruction loss works synergistically to achieve effective detection and isolation of unknown samples. This well-structured feature space directly supports the highest known class accuracy and AUROC values achieved by MTPL in the quantitative evaluations.

#### 5.5.4. Analysis of Model Component Effectiveness

To systematically evaluate the effectiveness of each component of our proposed MTPL framework, we conducted a series of ablation studies. By removing or replacing key components within the model and observing the changes in average recognition accuracy under different quantities of unknown classes (*N* = 1 to *N* = 6), we aim to quantify the contribution of each part to the overall performance of the complete MTPL model (as shown in Table 5).

First, the baseline model using only the classification loss (*L_c_* only) exhibits relatively low recognition accuracy across all values of *N*, reaching a maximum of only 89.84% for *N* = 1 and dropping significantly to 84.01% at *N* = 6. This indicates that classification loss alone is insufficient for learning features with strong discriminative power for numerous unknown devices, and the model’s robustness to unknown samples is inadequate, limiting its generalization capability.

Removing the classification loss setting (w/o *L_c_*) results in a drastic drop in recognition accuracy, with the value at *N* = 6 being only 18.75%. This result directly and strongly demonstrates the core role of classification loss in guiding the model to learn discriminative features for identifying known devices. Without direct classification supervision, even with the incorporation of reconstruction loss (*L_r_*) and prototype loss (*L_p_*), the model fails to effectively recognize known devices, thus failing to construct a functional recognition system. This clarifies that *L_c_* is fundamental to achieving effective recognition within the MTPL framework.

When the prototype loss setting is removed (w/o *L_p_*), the recognition accuracy from *N* = 1 to *N* = 5 improves (peaking at 93.66%), which is better than the baseline. However, compared to the complete MTPL method, its performance at *N* = 6 (88.48%) is significantly lower than MTPL’s 92.41%. This strongly proves the critical role of prototype loss in enhancing intra-class compactness and inter-class separability of features. It enables the model to maintain higher recognition accuracy and overall performance stability when facing a large number of unknown classes. Prototype loss effectively enhances the model’s resilience to unknown interference by constructing a more ordered feature space.

Removing the reconstruction loss setting (w/o *L_r_*), the performance is robust when the number of unknown classes is small (*N* = 1 to *N* = 3) but deteriorates significantly as the number of unknown classes increases, reaching only 90.34% at *N* = 6, which is notably lower than MTPL’s 92.41%. This validates the importance of reconstruction loss in preserving subtle signal variations and underlying structural information. This information is crucial for distinguishing unknown devices; its absence may lead to excessive generalization of model features, weakening the discriminative capability for unknown samples.

Regarding the discrimination mechanism, replacing it with KNN (w/o EVT with KNN) yields relatively good performance across all N*N* values (91.45% at *N* = 6) but is consistently 1–3% lower than the complete MTPL method across all N*N* values. This suggests that the EVT approach is superior to KNN in capturing the distribution characteristics of unknown samples and providing more robust and accurate decision boundaries. On the other hand, when replacing it with SVM (w/o EVT with SVM), performance degrades sharply from *N* ≥ 4, reaching only 78.48% at *N* = 6. This highlights the empirical nature of SVM’s decision boundaries, its insufficient generalization capability, and its poor adaptability to changes in the number of unknown classes, posing challenges for open-set recognition. In contrast, the statistically grounded decision framework provided by EVT is more stable and reliable.

## 6. Conclusions

Addressing the open-set recognition challenge in current RFFI techniques, this paper proposes an MTPL-based open-set recognition method. The core idea of this framework is to drive deep neural networks to learn a feature space that is “highly compact within classes and significantly separable between classes” through the joint optimization of three tasks: classification, reconstruction, and prototype learning. This approach establishes ideal conditions for subsequent open-set discrimination. Building upon this foundation, the method abandons traditional empirical thresholding and innovatively incorporates EVT to statistically model the distribution of prototype distances from known class samples, thereby enabling adaptive and robust determination of the decision boundary for distinguishing between known and unknown classes in open-set scenarios.

Extensive comparative experiments conducted on a public Wi-Fi radio frequency fingerprint dataset fully validate the effectiveness of the proposed method. Results demonstrate that MTPL achieves superior performance across various open-set test scenarios, particularly excelling in AUROC—a key metric for evaluating open-set recognition capability while maintaining the highest recognition accuracy for known classes. Furthermore, t-SNE visualizations intuitively confirm MTPL’s ability to construct a well-structured feature space, and ablation studies verify the necessity of the joint optimization of the three tasks.

Despite these promising results, this study has certain limitations. First, the current experiments are based on a medium-scale dataset; the generalization ability and computational efficiency of the method on large-scale datasets involving a greater number of devices require further systematic evaluation. Second, the research primarily focuses on Wi-Fi signals. Its generalizability to other radio frequency technologies, such as LoRa and 5G, as well as its robustness against channel distortions and noise in complex real-world environments, warrants more comprehensive experimental validation. Based on the above analysis, we will further pursue the following research directions: (1) construct large-scale heterogeneous radio frequency datasets to systematically evaluate MTPL’s performance under diverse communication protocols (Wi-Fi, LoRa, 5G), multipath conditions, and noise levels; (2) develop an incremental learning mechanism for dynamic networks by integrating few-shot and continual learning techniques with the structured feature space learned by MTPL, enabling online incremental authentication of new devices while mitigating “catastrophic forgetting” of existing knowledge; and (3) explore model light-weighting strategies, such as knowledge distillation and neural network pruning, to develop an edge-deployable version that maintains high recognition performance while reducing deployment cost and power consumption.

## Figures and Tables

**Table 1:** Network architectures of the encoder, decoder, and classifier.

Module	Structure	Layers
Encoder	ComplexConv (out_channels = 64, kernel_size = 3, stride = 2) ReLU + BN + MaxPool1d (kernel_size = 2)	×9
	Flatten + Linear (128)	×1
Decoder	Linear (128) + Resize	×1
	ComplexConv _trans (out_channels = 64, kernel_size = 3, stride = 2) ReLU + BN + MaxPool1d (kernel_size = 2)	×9
Classifier	Linear (128) + ReLU	×1
	Linear (Known_classes = 10)	×1

**Table 2:** Summary of baseline methods.

Method	Core Idea	Open-Set Discrimination Mechanism
SoftMax-T [23]	Standard cross-entropy classification.	Compares the maximum SoftMax probability against an empirical threshold.
OpenMax [24]	Calibrates the activation values of the SoftMax output layer.	Utilizes extreme value theory (EVT) to model the tail of activation scores for each class, calculating the probability of unknown classes.
MLOSR [28]	Combines classification and reconstruction tasks.	Uses extreme value theory (EVT) to model the tail of reconstruction errors for all known classes.
SR2CNN [33]	Multi-task learning combining classification, reconstruction, and center loss.	Compares the minimum distance between test samples and class centers against an empirical threshold.
TripletNet [35]	Metric learning based on triplet loss.	Compares the average distance between test samples and their k-nearest neighbors against an empirical threshold.

**Table 3:** AUROC comparison on the Wi-Fi dataset.

**Method**	*N* = 1	*N* = 2	*N* = 3	*N* = 4	*N* = 5	*N* = 6	Average
SoftMax	0.7436	0.7471	0.7515	0.6705	0.6624	0.7027	0.7129
OpenMax	0.9128	0.5898	0.4383	0.5787	0.6630	0.6728	0.6425
MLOSR	0.9138	0.9128	0.9101	0.9042	0.9142	0.8658	0.9034
SR2CNN	0.9465	0.9336	0.9363	0.9487	0.9438	0.9516	0.9434
TripletNet	0.9720	0.9782	0.9777	0.9822	0.9856	0.9523	0.9746
**MTPL (Ours)**	0.9968	0.9979	0.9986	0.9984	0.9986	0.9609	0.9918

**Table 4:** Open-set recognition accuracy (%) of different methods on the Wi-Fi dataset.

**Method**	*N* = 0	*N* = 1	*N* = 2	*N* = 3	*N* = 4	*N* = 5	*N = 6*
SoftMax	99.85	85.42	81.05	77.21	73.18	69.54	66.23
OpenMax	99.99	84.06	86.25	80.07	81.50	83.01	78.39
MLOSR	99.96	90.65	83.10	76.71	71.23	66.48	62.32
SR2CNN	99.99	75.70	77.64	79.36	78.69	80.08	77.43
TripletNet	99.98	96.00	96.33	96.53	96.78	97.00	91.48
**MTPL (Ours)**	**99.99**	**98.27**	**98.41**	**98.53**	**98.30**	**98.40**	**92.41**

Note: **Number** represents the top-1 accuracy.

**Table 5:** Recognition accuracy (%) under different model configurations.

**Model Configuration**	*N* = 1	*N* = 2	*N* = 3	*N* = 4	*N* = 5	*N* = 6
MTPL (Ours)	98.27	98.41	98.53	98.30	98.40	92.41
$L_{c}$ only	89.84	88.33	88.00	88.85	89.60	84.01
$w/o\;L_{p}$	91.75	92.43	92.90	93.21	93.66	88.48
$w/o\;L_{r}$	97.63	97.83	98.00	96.35	96.30	90.34
$w/o\;L_{c}$	27.27	25.00	23.07	21.42	20.00	18.75
w/o EVT (with KNN)	95.22	95.62	95.96	96.25	96.50	91.45
w/o EVT (with SVM)	95.88	96.22	96.51	89.67	83.70	78.48

## Data Availability

Not applicable.
